# LoRa Resource Allocation Algorithm for Higher Data Rates

**DOI:** 10.3390/s25020518

**Published:** 2025-01-17

**Authors:** Hossein Keshmiri, Gazi M. E. Rahman, Khan A. Wahid

**Affiliations:** Department of Electrical and Computer Engineering, University of Saskatchewan, Saskatoon, SK S7N 5A2, Canada; ehsan.rahman@usask.ca (G.M.E.R.); khan.wahid@usask.ca (K.A.W.)

**Keywords:** image transmission, LoRa PHY, LoRa MAC layer, LoRa modulation, SF selection

## Abstract

LoRa modulation is a widely used technology known for its long-range transmission capabilities, making it ideal for applications with low data rate requirements, such as IoT-enabled sensor networks. However, its inherent low data rate poses a challenge for applications that require higher throughput, such as video surveillance and disaster monitoring, where large image files must be transmitted over long distances in areas with limited communication infrastructure. In this paper, we introduce the LoRa Resource Allocation (LRA) algorithm, designed to address these limitations by enabling parallel transmissions, thereby reducing the total transmission time (*T_tx_*) and increasing the bit rate (BR). The LRA algorithm leverages the quasi-orthogonality of LoRa’s Spreading Factors (SFs) and employs specially designed end devices equipped with dual LoRa transceivers, each operating on a distinct SF. For experimental analysis we choose an image transmission application and investigate various parameter combinations affecting *T_tx_* to optimize interference, BR, and image quality. Experimental results show that our proposed algorithm reduces *T_tx_* by 42.36% and 19.98% for SF combinations of seven and eight, and eight and nine, respectively. In terms of BR, we observe improvements of 73.5% and 24.97% for these same combinations. Furthermore, BER analysis confirms that the LRA algorithm delivers high-quality images at SNR levels above −5 dB in line-of-sight communication scenarios.

## 1. Introduction

LoRa modulation is widely recognized for its long-range, low-power transmission capabilities, making it an attractive option for a broad spectrum of applications. Its efficiency and cost-effectiveness have made LoRa particularly suitable for use in cases that require low data rates, such as environmental monitoring, smart agriculture, and IoT networks. However, the low data rates inherent to LoRa present a challenge for applications that demand higher throughput, such as video surveillance, disaster monitoring, and plant phenotyping. These applications often require the transmission of large image files over long distances in areas where reliable communication infrastructure is lacking. Given its low complexity and energy efficiency, LoRa holds promise in such scenarios. Yet, the existing technology can result in prolonged transmission times, especially when dealing with high-resolution images or when operating under less-than-ideal channel conditions. To address these limitations, research has been focused on enhancing LoRa’s performance, aiming to increase its data rate while maintaining its key advantages.

In 2016, C. Pham [1] took the first step towards transmitting images, only a year after the birth of the LoRa framework. In this research, a low-cost, low-power image sensor is presented that implements an image compression technique to reduce the data size for long-range transmissions. Wei et al. [2] proposed an image transmission technique with various spreading factors (SFs) which utilized three separate nodes operating on different SFs to collaborate in sending images to their designated peers on the receiver side. Each LoRa node is controlled by a dedicated Raspberry pi and the data segments of the image are distributed among the senders using MQTT protocol. Chen et al. [3] proposed a lightweight and reliable communication protocol called MPLR, specifically designed for image dispatching over LoRa networks in an agricultural IoT platform. The MPLR protocol optimizes packet transmission by grouping information packets and utilizing bit-vector acknowledgments, which reduces the number of acknowledgment packets required and the associated wait times. To further minimize packet collisions caused by network congestion, MPLR employs a separate channel for transmitting request packets, distinct from the channels used for data and acknowledgment signals.

Achieving higher data rates in LoRa communications is also possible by upgrading the modulation technique. However, any modifications to LoRa modulation will render the existing modules incompatible with current applications and new hardware design will be required, resulting in increased complexity. In this paper, we have introduced the LoRa Resource Allocation (LRA) algorithm capable of parallel transmissions of the same data, reducing the total transmission time (*T_tx_*) and increasing the Bit Rate (BR). Inspired by the quasi-orthogonality of Spreading Factors (SFs) in LoRa [4,5], our proposed algorithm utilizes specially designed end devices that consist of two LoRa transceivers connected to a single controller unit, ensuring simple hardware implementation. For the experimental analysis, an image transmission application is chosen. We have investigated all possible parameter combinations affecting *T_tx_* to identify the optimal results in terms of interference, BR, and received image quality. Experimental results show that the proposed algorithm can significantly improve the *T_tx_* and BR compared to a typical LoRa framework for a Line of Sight (LoS) communication link while maintaining a high image quality. The main contributions of this research are as follows:

1. Proposes a specially designed end device (DED) to practically evaluate the quasi-orthogonality feature of LoRa in SF and RF channels and suggests optimal SF combinations for parallel transmission;
2. Proposes a lightweight, synchronized resource-allocation algorithm for transmission time and bit rate improvement;
3. Derives mathematical models for the proposed algorithm regarding SF allocation for the total parallel transmission time, data rate, and energy consumption;
4. Implements the identified SF combinations and evaluates the throughput improvement;
5. Gives insight about the Bit Error Rate (BER) performance of the parallel setup and the quality of the images received.

The rest of the paper is organized as follows. In Section 2 we have presented a literature review of the related studies in the field. Section 3 illustrates in detail the framework of the proposed algorithm. Section 4 presents the experimental study and the results are presented. Finally, Section 5 concludes the paper by summarizing the results and suggesting directions for future research.

## 2. Related Work

Recent studies in the literature have explored various methods to enhance the data rate of LoRa by modifying its Chirp Spread Spectrum (CSS) modulation. Edward et al. [6] proposed the use of Interleaved Chirp Spreading (ICS) alongside traditional chirp spreading. This technique incorporates an additional bit into the LoRa symbol for ICS, leading to a 42% increase in capacity. Building on this concept, a Slope Shift Keying (SSK) approach with ICS was introduced in [7], which utilizes a combination of up, down, and interleaved up-and-down chirp modulation. This method further boosts the data rate, achieving a 28.6% improvement compared to the standard CSS LoRa with the same SF and BW. Kang [8] proposed a new index modulation for LoRa that utilizes multiple quasi-orthogonal chirps modulated under different SFs, enabling it to carry more information compared to the conventional LoRa. Hanif et al. [9] introduced the frequency shift chirp spread spectrum modulation that exploits index modulation (FSCSS-IM) to increase the data rate. Instead of one chirp at a time, FSCSS-IM transmits multiple chirps simultaneously. In [10], Time-Domain Multiplexing (TDM) is employed for LoRa modulation, distributing bits of a symbol across different SFs. This method doubles the data rate with a minor degradation in BER, particularly at the lower SF of 7. Additionally, in [11], authors introduced a data rate enhancement approach known as PATCH (Phase-based Additional Channel), which utilizes the phase of the chirp signal as an additional channel. This technique can boost the BR by up to 28.57% for SF = 7. Nurbay et al. [12] introduced a cooperative framework for transmitting large files over LoRa. They developed an algorithm that leverages neighboring nodes, selecting them based on their SF and packet success rate to share portions of the data. To facilitate communication between nodes, the framework utilizes a high data rate medium like Wi-Fi for exchanging algorithmic messages among the neighbors.

Some research efforts have proposed enhancements to the network access techniques for the LoRa communication protocol. For instance, a dynamic spreading factor allocation algorithm was introduced in [13] to maximize the data rate for the communication link. This algorithm optimizes the data rate by considering the hop count, ensuring that higher SFs are assigned to links with fewer hops or clusters that are closer, thereby equalizing the packet travel time or Time on Air (ToA). The approach is capacity-focused, utilizing an iterative process to allocate SFs, enhancing overall network performance.

The LoRaWAN Gateway (GW) is not full-duplex, and relies on an Adaptive Data Rate (ADR) to adjust the SF. However, this adjustment becomes impractical when the GW is mobile at speeds above 2 km/h. In such scenarios, the GW must frequently switch SF and channels to communicate with end devices (EDs), leading to significant overhead and reducing goodput to as low as 20%. To address this, Cantor [14] was implemented in a single-GW LoRaWAN network to gather network parameters from EDs, optimize the Packet Reception Rate (PRR), and estimate the actual downlink (DL) PRR using an optimization algorithm. By employing a regression model (WW: Wane and Wax, representing alternate increase and decrease), Cantor more accurately determines PRR and improves goodput by up to 70%. Additionally, Cantor’s use of windowing for acknowledgments (ACK) shows better performance with higher SFs.

The distance-based SF allocation using the Exponential Windowing Scheme (EWS) [15] is designed to reduce co-SF interference and improve network capacity. This approach utilizes an offline optimization algorithm, making it particularly effective for static EDs. The method achieves a PDR improvement of 18.2% to 55.25% compared to similar SF allocation algorithms. However, a significant drawback is the exponential decrease in data throughput as the number of EDs increases from 1000 to 8000. Additionally, EWS requires GPS-based location data, RSSI-based distance calculation, or manual distance measurement, adding complexity to its implementation. In contrast, the proposed LRA algorithm avoids offline calculations to maintain a lightweight protocol. Kang et al. [16] proposed Multiple-Input Multiple-Output (MIMO)-LoRa, which enhances BER at higher SFs of 10, 11, and 12 by utilizing multiple SFs for parallel transmission. This approach employs a precoding matrix to optimize the total SNR and peak transmit power to select the appropriate SFs.

The LoRa GW chip is designed to decode superimposed signals from different SFs, as proposed in [17]. The demodulator is divided into two groups, handling odd and even SFs separately. To efficiently utilize the MAC layer, which treats all packets from different SFs as a single transmission, strict synchronization across all EDs is required. However, using higher SFs increases the likelihood of network congestion. Since higher SFs result in longer transmission times, they can occupy the channel for extended periods, potentially leading to congestion in networks with multiple devices. As a result, it may be necessary to classify EDs to manage the SF-based packet distribution effectively and avoid congestion-related issues. Some research works have focused on lowering the size of the image by employing encoders and compression techniques to reduce the total data size required for the image transmission [18,19].

Some other works adopted machine-learning-based strategies for dynamic SF allocation. Scarvaglieri et al. [20] proposed an energy-efficient resource allocation strategy based on Q-Learning, enabling LoRa devices to make autonomous decisions on SF configurations. However, as Q-Learning algorithms require multiple iterations to converge to optimal decisions, in dynamic environments where conditions like interference and device density change frequently, this convergence time could limit performance. Ta et al. [21] focused on mitigating inter-SF interference using a Multi-Armed Bandit (MAB) model and EXP3 (Exponential Weights for Exploration and Exploitation) algorithm, enabling LoRa devices to autonomously select the least-congested SFs. Although the EXP3 algorithm is computationally efficient, it may require time to converge on optimal SF allocations, which could lead to inefficiencies during periods of high network changes or initialization phases.

A Deep Reinforcement Learning (DRL)-based SF optimization algorithm that dynamically adjusts SF by considering both node characteristics and channel conditions is proposed in [22]. The algorithm uses retransmission as a proxy for detecting collisions. This indirect method may introduce inaccuracies in environments where packet losses occur due to interference or poor signal conditions rather than collisions. Busacca et al. [23] introduced FedLoRa, a federated learning-based optimization scheme that balances resource allocation to minimize FML (Federated Machine Learning) round time and energy consumption by distributing network load across available SFs. A greedy algorithm is proposed to approximate the optimization problem of resource allocation.

## 3. LRA Algorithm Framework

### 3.1. Physical Layer

LoRa uses CSS modulation at various ISM bands such as 915 MHz for North America. LoRa transceivers, such as the SX1276, support programmable bandwidths (BW) from 7.8 to 500 kHz. Some of the most used BW settings are 125 kHz, 250 kHz, and 500 kHz. SF represents the number of bits carried by each symbol and is directly proportional to the symbol time or chirp duration. Longer SFs result in longer symbol times and vice versa. Signals modulated with different SFs are said to be quasi-orthogonal [4], thus they can co-exist and be decoded successfully at the same time. The quasi-orthogonality can be proved by following the principle of orthogonal functions. Assume that $s_{n}(t)$ is the time domain representation of a chirp signal with SF=n.

(1) $$s_{n}(t)=Ae^{j2\pi (f_{0}t+\frac{B}{2T_{s,n}}t^{2})}$$

*B* is the BW, $T_{s,n}$ is the symbol time, and $f_{0}$ is the frequency offset that refers to the initial frequency of the chirp for SF=n and are defined as below:

(2) $$T_{s,n}=\frac{2^{n}}{B}$$

(3) $$f_{0}=\frac{B}{2^{n}}k$$

where $k\in \{0,\ldots ,2^{n}-1\}$ is the chip decimal value. Two signals with SF=n and SF=p, n≠p are quasi-orthogonal if their inner product is near zero.

(4) $$<s_{n}\left(t\right).s_{p}^{*}\left(t\right)>=\int s_{n\left(t\right)}s_{p}^{*}\left(t\right)dt≅0$$

Integral in (4) can be expanded as (5).

(5) $$A^{2}\int e^{j2\pi \left[\left(f_{0,n}-f_{0,p}\right)t\right]}dt+A^{2}\int e^{j2\pi \left[\left(\frac{1}{T_{s,n}}-\frac{1}{T_{s,p}}\right)\frac{B}{2}t^{2}\right]}dt$$

Integrating the first term over interval [0,T], $T=max(T_{s,n},T_{s,p})$ is zero. The phase of the second term is generally a large value (61.0352 × 10^6^ for B=125 kHz, n=7, p=8) causing rapid oscillations throughout the interval. Using the stationary phase approximation will yield the magnitude of √(π⁄a) where $a=2\pi (1/T_{s,n}-1/T_{s,p})B/2$, indicating a near-zero value for the inner product.

The actual BR depends on the SF, Coding Rate (CR), and BW, and is calculated using (6) [24], which gives a maximum BR of 21.875 kbps for BW = 500 kHz, CR = 1, and SF = 7.

(6) $$BR=SF\frac{4}{4+CR}\frac{B}{2^{SF}}$$

The transmission power supported by LoRa is adjustable from −4 dBm to +20 dBm. The lowest receiver sensitivity is around −130 dBm, and it performs better at higher SF and lower BW (−136 dBm at SF = 12 and BW = 125 kHz) [24]. This sensitivity increases LoRa’s range at higher SF and lower BW over other LPWAN technologies [25]. The range of LoRa transmission depends on the radio link conditions and the antenna height to reduce signal reflection and absorption by the ground.

**Figure 1:**
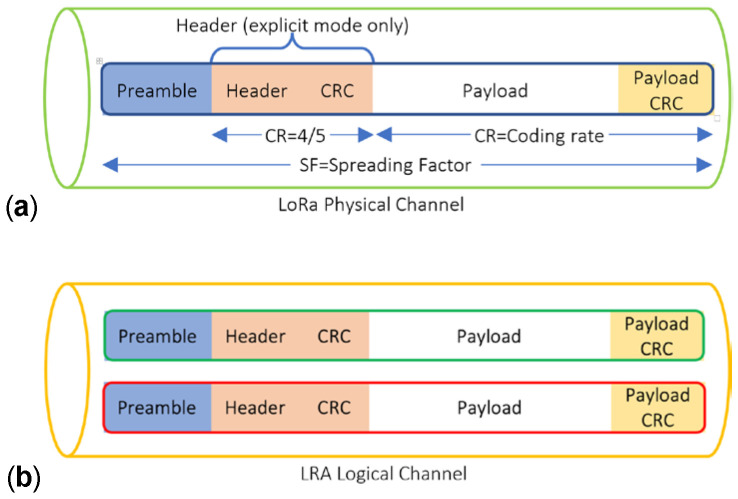
LoRa structure. (**a**) Physical channel, and (**b**) logical channel used in LRA algorithm.

Keeping the current LoRa modulation unchanged, its capacity in terms of the BR can be increased with parallel transmissions and receptions of the payload. Unlike the GW LoRa modem (such as SX13XX or similar), the existing LoRa EDs do not support multiple channels and SFs for simultaneous communication. However, LoRa link capacity and network goodput can be improved by utilizing its orthogonality features and by improving the MAC protocol. As shown in Figure 1a, the LoRa physical layer packet consists of two mandatory blocks called the Preamble and Payload, along with two optional blocks for the header and Payload-CRC (Cyclic Redundancy Check). Figure 1b shows our proposed parallel setup by grouping two LoRa physical channels. These channels have the same configuration (BW, RF and CR), except for the different SFs, and are highly synchronized to transfer the data stream.

Due to the similarity of configuration and operation, in the rest of this paper this channel is called a logical channel (LC), to facilitate describing the proposed method. All the block sizes are variable according to the transceiver configuration and the payload size ranges from 1 to 255 bytes.

### 3.2. Designed End Device (DED)

**Figure 2:**
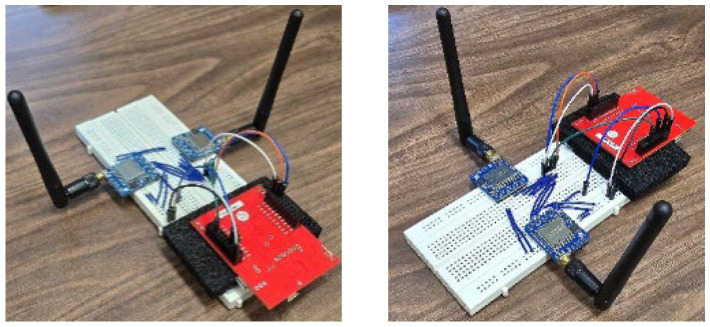
Lab prototype of the DED.

Based on the proposed LC (Figure 1b), a customized end device is designed that comprises two Semtech SX1276 LoRa transceivers connected and controlled by a single processor to avoid data processing/sharing complexity. As shown in Figure 2, the processor is a Texas Instrument Tiva C series unit and is programmed using Energia IDE 1.8.11, a fork of Arduino IDE. Both LoRa modules are connected to a shared Synchronous Peripheral Interface (SPI) bus and are equipped with identical dipole antennas for enhanced transmission power. The LRA algorithm, which is elaborated in the next section, is performed on this processor and the resulting data packets are fed to the radios to be transmitted simultaneously.

**Figure 3:**
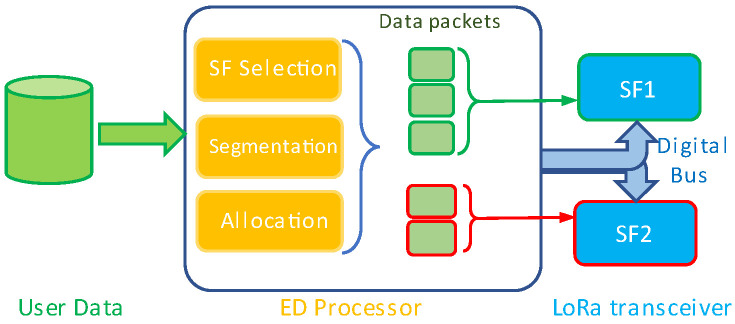
DED’s block diagram for the LRA algorithm implementation.

It is assumed that multiple SFs are used in the same RF channel, which are not assigned for any other link at the same time in the network to avoid co-channel interference. Figure 3 shows the hardware and software blocks of the DED with two LoRa transceiver modules used for the proposed LRA algorithm implementation. All the transceivers are configured to have the same LoRa parameters, except for SF. The transferred data are measured in bytes.

**Figure 4:**
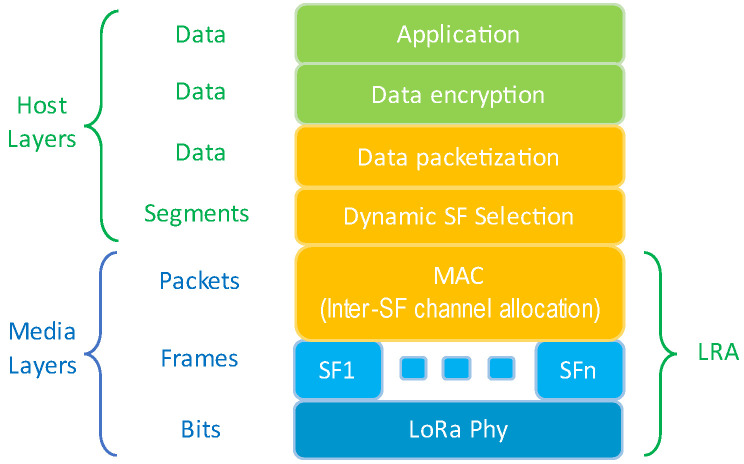
Proposed LoRa stack for the LRA algorithm.

To improve network capacity, the LRA algorithm improves the BR of a LoRa link and the goodput of the network by reducing the total data transmission time *T_tx_* between EDs and GW, regardless of the application type. As shown in Figure 4, the physical layer utilizes the LoRa SF orthogonality for parallel data transmission by efficiently selecting multiple SFs. The proposed algorithm can act as a MAC layer that facilitates packet distribution and synchronization among the SFs.

### 3.3. Resource Allocation Algorithm

**Figure 5:**
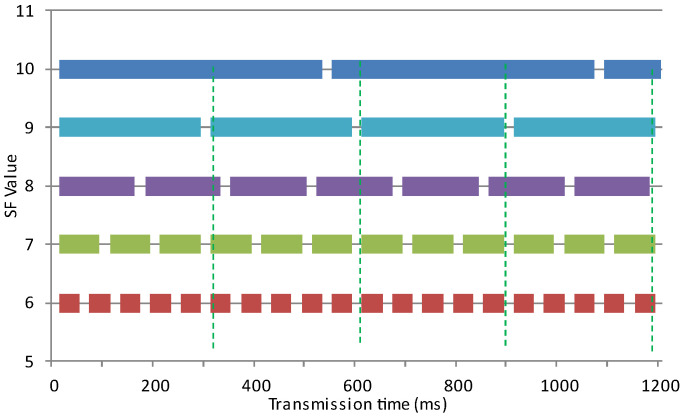
ToA of burst transmission of 240 B packets over different SF with the same RF channel and BW.

Based on the achievable BR for a given payload size, the ToA related to each SF can be derived. Assume that packets with the equal payload of 240 bytes are to be successively transmitted. Figure 5 shows the transmission times of 240-byte packets using different SFs, with each block representing the time of a single packet. The gaps between the blocks are packet processing times ($t_{u}$). In this example, the transmission takes about 300 ms if SF = 9, while it only takes approximately 60 ms for SF = 7. SF = 6 is not considered in our algorithm due to its unavailability in some transceivers. As indicated by the green dashed lines, packets transferred using SF = 7 achieve better synchronization with packets transferred concurrently using SF = 9, compared to those using SF = 8 or SF = 10. Therefore, synchronous access of LC can be maintained by allocating different portions of data with equal packet sizes to the physical channels.

The proposed algorithm assigns a specific number of data packets sequentially to the transmitters based on the amount of data they can send through the LC. As illustrated in Figure 5, while transmitting a single packet at SF = 9, it is possible to transmit three packets at SF = 7. However, by introducing a slight delay in packet processing, the system can transmit two packets at SF = 8 alongside three consecutive packets at SF = 7, contributing to a higher BR compared to other SF combinations. In other words, three out of five packets are sent by the transmitter with SF = 7 and two are sent by the one with SF = 8 per each round of parallel transmission. Thus, to maximize channel utilization and minimize the overall transmission time, the algorithm allocates 60% of the entire user data to SF = 7 and the remaining 40% to SF = 8. The pseudocode of the proposed algorithm is shown in Algorithm 1.
**Algorithm 1.** Pseudocode for LRA algorithm.//Synchronized Resource Allocation Algorithm //For a logical channel that contains 2 physical channels with spreading //factors SF1 and SF2 and SF1 < SF2 //Assumptions: //All parameters (packet sizes, SFs, BW, CR, etc.) are provided by //the user //Variables m = size(input_byte_string); p = packet_size; N = floor(m/p) + 1; //number of all packets Delay;       //delay time //Initialize: Set parameters for the transmitters  Based on the measured ToAs (Figure 5) for SF1 and SF2, determine:  n1 = number of all packets for SF1  n2 = number of all packets for SF2 (n2 = N − n1) //Loop until all packets are transmitted For (i = 1:N, i++) {  Transmit using SF1;  //to complete n1 packets  Transmit using SF2;  //to complete n2 packets  //delay after each parallel transmission to allow packet processing  //on the receiver  Delay;} }

### 3.4. SF Selection

SF represents the number of chips per chirp or symbol; thus, as the SF grows, the symbol duration increases. In other words, higher SF leads to lower BR and higher ToA. Therefore, maximum BR depends on the ToA, which is calculated based on the BW and SF, as shown in (7) [24].

(7) $$ToA=\left(20.25+\left\lceil \frac{2PL-SF+7}{SF}\right\rceil 5\right)\left(\frac{2^{SF}}{B}\right)$$

ToA will be the lowest for all the SFs where BW is the highest. Table 1 shows the ToA for BWs 250 kHz and 500 kHZ when SF is varied, and the relative BR improvement ($RBRI=1-{BR}_{i}/{BR}_{i+1}$) between two consecutive SFs for the same BW. It also indicates that the ToA with BW = 250 kHz and SF = 7 is only 20.4 ms (12%), less than the ToA with BW = 500 kHz and SF = 8. This differs more (55% to 57%) for the same BW with different SF. Therefore, parallel transmission using 250 kHz and 500 kHz BW can be used for two consecutive SF values. However, it is not used in the proposed LRA algorithm to avoid data loss due to inter-channel interference, as further explained in Section 4.

### 3.5. LRA Algorithm Data Rate

The actual data rate for the proposed LRA algorithm depends on the equivalent or total ToA for the parallel channels. Total ToA, which is the sum of the travel times of all packets using different SFs, can be represented by (8):

(8) $${ToA}_{tx}=\sum_{m=1}^{3}\sum_{n=0}^{6}{ToA}_{m,n}=\sum_{m=1}^{3}\sum_{n=1}^{6}{ToA}_{m,0}{(\alpha }^{-n}\beta^{-m})$$

where *m* is the index of different BWs used in the range of 125 kHz to 500 kHz, and *n* is the index of different SF values used from 6 to 12. *ToA_m_*_,0_ is the ToA at SF = 6 for a specific BW. Table 1 shows that the ToA is the lowest at SF = 6 for a specific BW.

**Figure 6:**
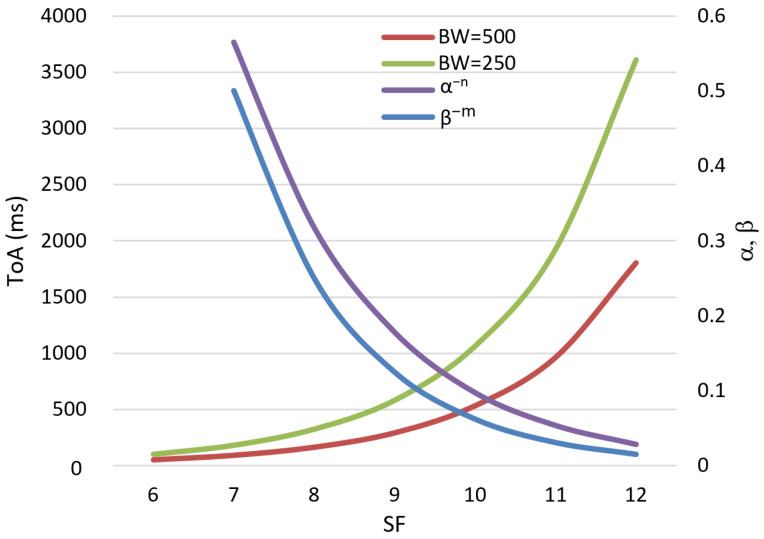
Impact of changing BW and SF on ToA.

The increment of the ToA for another SF is represented by *α*. The ToA increment for the same SF at a different BW is represented by *β*. Figure 6 shows the variation in ToA for different SFs and BWs, from which we can determine the values of *α* and *β*, which vary for different SFs and BWs. However, these variations are minimal, about 10%. Therefore, an approximation is used where *α* = 0.56 and *β* = 0.5.

Burst data transmission using the proposed algorithm requires multiple LoRa packets to be transmitted sequentially. This packet sequence can transmit a data stream in multiple time slots, as performed in TDMA, due to the different ToAs at different SFs. The number of packets for a burst transmission may also induce further underutilization. This utilization can be maximized using odd or even SF values for the stream of data packets, which corresponds to either SF = 7 and SF = 9, and SF = 8 and SF = 10 being paired together. However, it depends on the data processing and the SPI communication time, which are almost fixed for all SFs. Therefore, odd or even paired SFs may not be the best choice for maximum resource utilization.

For the burst transmission of multiple LoRa packets, the total burst transmission time *T_tx_* includes the total ToA (*ToA_tx_*) and the total processing time (*T_u_*), as shown in (9). However, the number of packets (*F*) that need to be transferred will differ for different SFs in an LC. Considering the same processing time for all the SFs, *ToA_tx_* and *T_u_* can be rewritten as (10) and (11). From Figure 5, it can be shown that the synchronization loss will be the lowest when the ToA is the minimum time required for a packet, which is *ToA_n_* − *t_u_*, and that the transmission time utilization can be expressed by (12). The lowest ToA occurs for the lowest SF used, which is seven for *n* = 1. Therefore, the synchronization loss can be minimized by using the smallest SF possible and transmitting the largest data segment using that SF. The effective BR of the proposed algorithm can be calculated using (13), using *PL* as the payload size in bits and *T_tx_* in seconds. $t_{u}$ is the delay time required for packet processing and reception at the receiver.

(9) $$T_{tx}={ToA}_{tx}+T_{u}$$

(10) $${ToA}_{tx}=\sum_{m=1}^{3}\sum_{n=1}^{6}{F_{m,n}ToA}_{m,0}(\alpha^{-n}\beta^{-m})$$

(11) $$T_{u}=t_{u}\sum_{m=1}^{3}\sum_{n=1}^{6}F_{m,n}$$

(12) $$\varphi =1-\frac{{ToA}_{n}-t_{u}}{T_{tx}}\approx 1-\frac{{ToA}_{1}-t_{u}}{T_{tx}}$$

(13) $${BR}_{LRA}=(1-\varphi )\frac{PL}{T_{tx}}$$

### 3.6. Energy Consumption Model

The energy consumption of the LRA algorithm can be modeled by the total energy consumed by DEDs. This total energy can be determined by calculating the energy needed for transmitting on each radio (specific SF and BW), the energy spent in standby mode to allow packet reception and processing at the receiver, and the energy required for receiving and processing packets. Equation (14) shows the total energy dissipated across all nodes. As shown in Equations (15) and (16), $E_{tx}$ is the energy required for burst data transmission, where $P_{tx}$ and $P_{s}$ are the LoRa transmit and standby power, respectively. $T_{u}$ is the sum of packet processing times during which the nodes are in standby mode. Equations (17) and (18) present the energy required for packet reception and processing, $E_{rx}$. The power required for the LoRa transceiver (such as SX1276) to transmit and receive is the same for all BWs and SFs. Therefore, $P_{tx}$ and $P_{rx}$ can be replaced with $P_{0}^{tx}$ and $P_{0}^{rx}$. These values are set as follows: $P_{0}^{tx}=287.1\;mW$, $P_{0}^{rx}=37.95\;mW$, and $P_{s}=5.28\;mW$ [24]. The total energy consumed can be represented as (19).

(14) $$E_{total}=E_{tx}+E_{rx}$$

(15) $$E_{tx}=P_{tx}{ToA}_{tx}+P_{s}T_{u}$$

(16) $$E_{tx}=P_{0}^{tx}\sum_{m=1}^{3}\sum_{n=1}^{6}F_{m,n}{ToA}_{0}\left(\alpha^{-n}\beta^{-m}\right)+P_{s}t_{u}\sum_{m=1}^{3}\sum_{n=1}^{6}F_{m,n}$$

(17) $$E_{rx}=P_{rx}{ToA}_{tx}+P_{s}T_{u}$$

(18) $$E_{rx}=P_{0}^{rx}\sum_{m=1}^{3}\sum_{n=1}^{6}F_{m,n}{ToA}_{0}\left(\alpha^{-n}\beta^{-m}\right)+P_{s}t_{u}\sum_{m=1}^{3}\sum_{n=1}^{6}F_{m,n}$$

(19) $$E_{total}=\left(P_{0}^{tx}+P_{0}^{rx}\right)\sum_{m=1}^{3}\sum_{n=1}^{6}F_{m,n}{ToA}_{0}\left(\alpha^{-n}\beta^{-m}\right)+2P_{s}t_{u}\sum_{m=1}^{3}\sum_{n=1}^{6}F_{m,n}$$

## 4. Experimental Study and Results

At the time of the writing of this manuscript, no LoRa modules capable of parallel transmission were found. The experimental study is organized in two phases. In the first phase, the functionality of our DED and resource allocation algorithm is investigated by conducting three distinct experiments using some small bytes as pilot data. Once the optimal set of parameters is found, the second phase is performed by sending image data and reporting the *T_tx_*, BR, BER, consumed energy, and received image quality.

### 4.1. Phase I

In these experiments we have investigated parallel transmission of the pilot data using:

1. Different radio channels with the same BW and SF;
2. Different BWs with the same radio channel and SF;
3. Different SFs with the same BW and radio channel.

**Figure 7:**
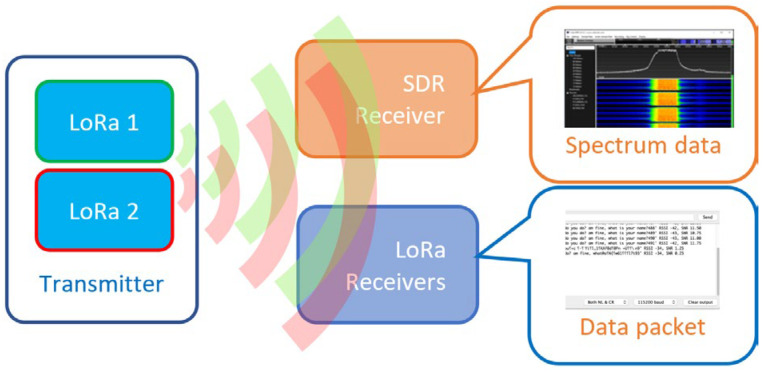
LoRa parallel data transfer experimental setup.

Since the third experiment with different SFs has already been thoroughly investigated in the literature (such as [26,27]), we chose not to present the analysis results for it to avoid redundancy. While varying the experimental parameters as required during the experiment, the remaining LoRa parameters were kept constant. The radio spectrum is monitored via a Software Defined Radio (SDR) receiver. The received data quality, RSSI and SNR of the transmitted signal were monitored using another DED, identical to the one at the transmitter, configured for specific SF, BW, and radio channels. The experimental setup is shown in Figure 7.

#### 4.1.1. Different Radio Channels

**Figure 8:**
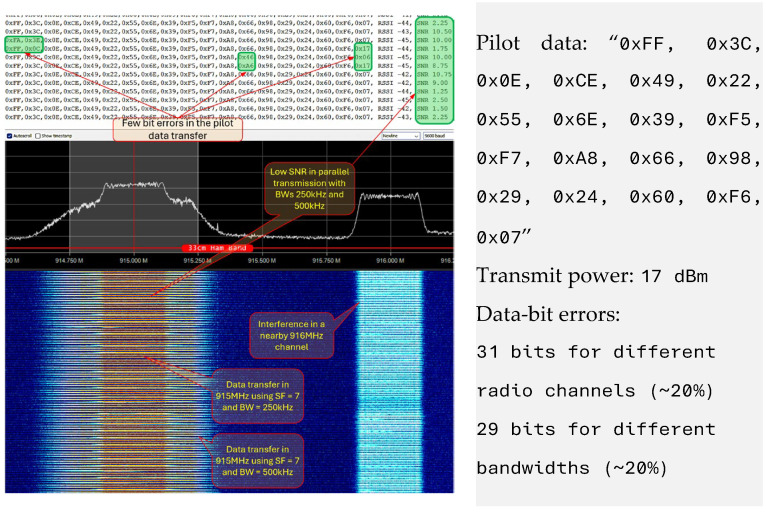
Different LoRa bandwidth and channel interference: The reflected radio channel (916 MHz) monitored for inter-channel interference. Data loss for LoRa parallel data transmission using two different BWs (BW 250 kHz and 500 kHz) with same SF = 7 and radio channel 915 MHz.

Inter-radio channel interference was monitored to determine the best radio channels for parallel data transmission. In this experiment, the receiver was configured with SF = 7, BW = 250 kHz, and the radio channel frequency of 915 MHz. The transmitter was configured with different radio channel frequencies ranging from 900 MHz to 940 MHz; BWs of 125 kHz, 250 kHz and 500 kHz; and SF values from 7 to 12. The radio channel was monitored using the spectrum analyzer, which showed more than one reflected channel other than the transmitted channel. This caused interference with the configured radio channel. Figure 8 shows a reflected radio channel at 916 MHz for the transmitted signal using a 915 MHz radio channel with BW = 250 kHz and SF = 7. Although the reflected channel signal level was lower compared to the actual channel, the receiver configured for the reflected channel received 5–10% of the packets with 20% data-bit loss. Therefore, it was determined that multiple LCs with different radio frequencies could not be used for parallel transmission.

#### 4.1.2. Different Bandwidths

In this experiment, one transmitter was configured to transmit 240 bytes of data using the 915 MHz radio channel with BW = 250 kHz and SF = 7. Another transmitter was configured to transfer a 240-byte packet using the same radio channel with SF = 7 and the BWs 125 kHz, 250 kHz, and 500 kHz. The receiver was configured using the 915 MHz radio channel with SF = 7. Figure 8 shows the parallel transmission of different BWs with the same SF and radio channel. The receiver received the data from the transmitter of a similar configuration (915 MHz, BW = 250 kHz, and SF = 7). However, the transmitted data with a BW of 500 kHz causes noise that affects the physical channel with a 250 kHz BW, resulting in an SNR decrease from 10 to 1.25. We also observed a few bits of data loss in 20% of the overlapped data packets, denoted by green boxes in Figure 8. Therefore, LCs of different BW could not be used for parallel transmission.

#### 4.1.3. Different Spreading Factors

In this experiment, the two transmitters were configured with the same parameters, except for SFs transmitting 240 bytes over the 915 MHz radio channel. As expected, due to the quasi-orthogonality of the SFs, no significant data loss was observed during parallel transmission when using SF = 7 in conjunction with SFs ranging from 8 to 10. However, in small SF combinations with higher SF values such as 7 and 12, the SNR value experienced a meaningful decrease. As different SFs are not perfectly orthogonal, this issue stems from inter-SF interference caused by the extended overlapping duration of the two transmission chirps. To mitigate this, long-duration overlaps should be avoided during parallel transmission using different SFs.

### 4.2. Phase II

In this section, the parallel transmission of image data using different SFs is examined in terms of *T_tx_*, BR, BER, energy consumption, and received image quality. To do this, small sized images (around 20 to 30 kB) are transformed into strings of bytes and stored in an SD RAM, connected to the processor. The communications were conducted in an open field spanning approximately 200 m, with both the transmitter and receiver positioned 3 m above the ground, ensuring a LoS link. On the transmitter and receiver, one LoRa radio is set to operate on SF = *i* and the other on SF = *j*, where *i*, *j* = {7, 8, 9, 10} and *i* ≠ *j*, to maximize the utilization of the LC, as discussed earlier in Section 3.3. CR and BW are the other parameters that contribute to the ToA (transmission time of one packet) and thus *T_tx_*. While a larger CR improves the robustness of the transmission against interference, it takes up packet capacity due to the added redundancy, thus diminishing the throughput of the LC and increasing the ToA. Therefore, CR in all experiments is fixed at 1 for all LoRa modules. We have repeated the experiment for every BW and measured the impact on *T_tx_*. The payload size of all LoRa modules is set to the highest value, 255 bytes.

The image bytes are treated as a pool of information from which packets are formed and sent. To send an image over the LC, the LRA algorithm extracts the corresponding image bytes from the pool and segments them based on the SFs used by the LoRa modules at the transmitter (Section 3.3). In the first trial, the SFs on the transmitter and receiver are set to 7 and 8. According to Figure 5, to minimize synchronization loss in this parallel setup, a delay should be introduced to allow the reception of 2 packets over SF = 8 and 3 packets over SF = 7.

This results in a total of 5 packets being received in a single round of parallel transmission, with 2 of them delivered using SF = 8. Table 2 shows the results for the total parallel transmission time of a 24 kB image over the LC. The delay column represents the minimum time required to process received packets on both LoRa modules, ensuring no packets are missing. This delay time is determined through repeated transmissions using the same parameter set.

As shown in (6), a higher BW results in a higher BR, which in turn reduces the *T_tx_*. The fastest image transfer was achieved with a BW of 500 kHz. This experiment was repeated for various combinations of SFs, with the corresponding *T_tx_*s reported in Table 3. The LRA algorithm allocates data to ensure a balanced packet distribution between the two transmitters for various SF combinations. The proportions for each SF combination are as follows. For SFs 7 and 9, 75% of the image data is allocated to SF = 7, and 25% to SF = 9. For SFs 7 and 10, 85% is allocated to SF = 7, and 15% to SF = 10. For SFs 8 and 9, 67% of the data is assigned to SF = 8, and 33% to SF = 9. Lastly, for SFs 8 and 10, 75% is allocated to SF = 8, and 25% to SF = 10.

In the next trial, the total transmission times using our proposed algorithm is compared with a single transceiver setup. In this regard, the same experiment is repeated using a single LoRa module at the transmitter and receiver operating on SF = 7. This time, the whole string of bytes is formed into equal sized packets and fed into the buffer of the transmitter. Table 4 shows the *T_tx_* for different BWs. In this case, the packet processing times are lower than the parallel transmission as no further delay is required for synchronizing a concurrently received packet. However, the overall transmission times are significantly longer compared to specific parallel SF combinations.

The underlying reason is the synchronization and distribution of data packets during parallel transmission. The proposed LRA algorithm assigns dedicated data segments to each LoRa radio in a synchronized manner, enabling simultaneous packet transmissions. This approach not only facilitates multiple transmissions at once but also reduces the total number of packets sent over each LoRa channel, ultimately leading to lower overall transmission times.

**Figure 9:**
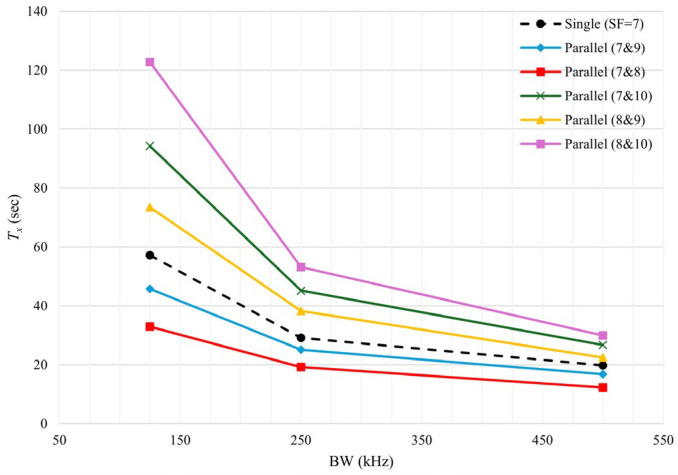
Transmission time for sending the same image over different parallel setups compared with a single transceiver setup.

**Figure 10:**
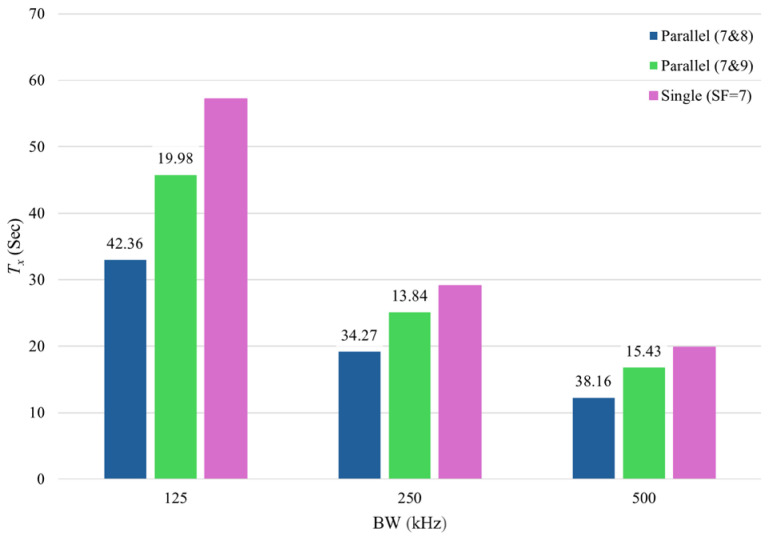
*T_x_* improvement of parallel setup compared to single setup.

Figure 9 demonstrates the effect of different SF combinations on the *T_tx_* compared to a single setup operating solely on SF = 7. In a single transceiver setup, the fastest transmission occurs with SF = 7, excluding SF = 6 due to its unavailability in some modules. As shown in Figure 9, the *T_tx_* can be significantly improved using SF combinations of 7 and 8 or 7 and 9. Other SF combinations fail to enhance *T_tx_*, as higher SFs lead to increased transmission times. The percentage of improvement for each parallel setup is detailed in Figure 10. The combination of SFs 7 and 8 improved the *T_tx_* by 42.36%, 34.27%, and 38.16% for BWs of 125 kHz, 250 kHz, and 500 kHz, respectively. Meanwhile, the parallel setup with SFs 7 and 9 achieved improvements of 19.98%, 13.84%, and 15.43% for the same BWs.

**Figure 11:**
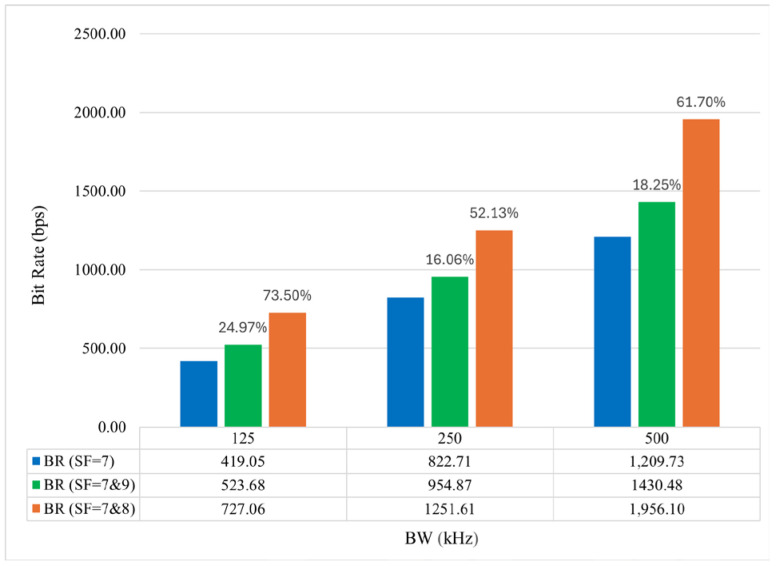
Bit rate improvement of parallel setup compared to single setup.

The performance of SF combinations that enhance *T_tx_* compared to a single transceiver setup is evaluated in terms of BR. Figure 11 presents the BR of a single LoRa channel operating on SF = 7, as well as LCs utilizing SF combinations of 7 and 8 and 7 and 9, while accounting for packet processing times. When SF = 7 and 8 are employed, the BR increases by 73.5%, 52.13%, and 61.7% for BWs of 125 kHz, 250 kHz, and 500 kHz, respectively. For the SF = 7 and 9 combination, the BR improvement is 24.97%, 16.06%, and 18.25% across the same BWs.

**Figure 12:**
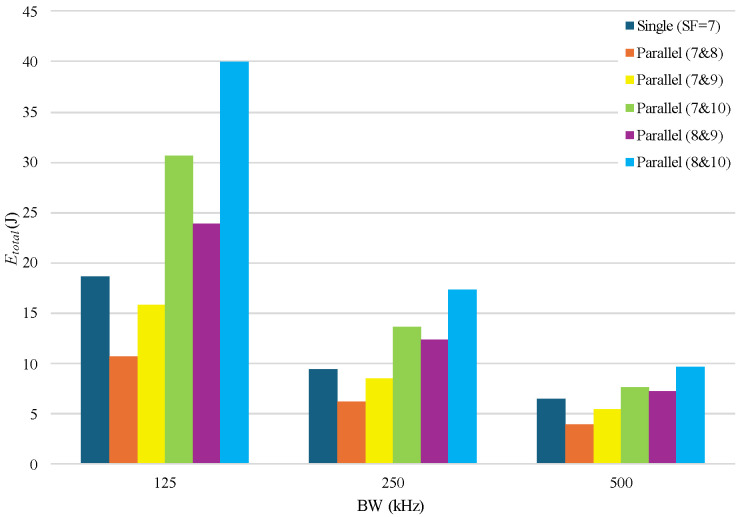
Comparison of total energy consumption of proposed LRA algorithm with single transceiver setup with SF = 7.

Figure 12 illustrates the energy consumption profile of our parallel setup using the proposed LRA algorithm, based on Equations (14)–(18). The transmitter amplifier and the receiver low noise amplifier consume the largest portion of the total energy. However, SF combinations 7 and 8, and 7 and 9 significantly reduce transmission time, allowing the amplifiers to remain active for shorter durations compared to a single transceiver setup. As a result, the LRA algorithm demonstrates better energy efficiency than the traditional single setup for SF combinations 7 and 8, and 7 and 9 across all BW values.

**Figure 13:**
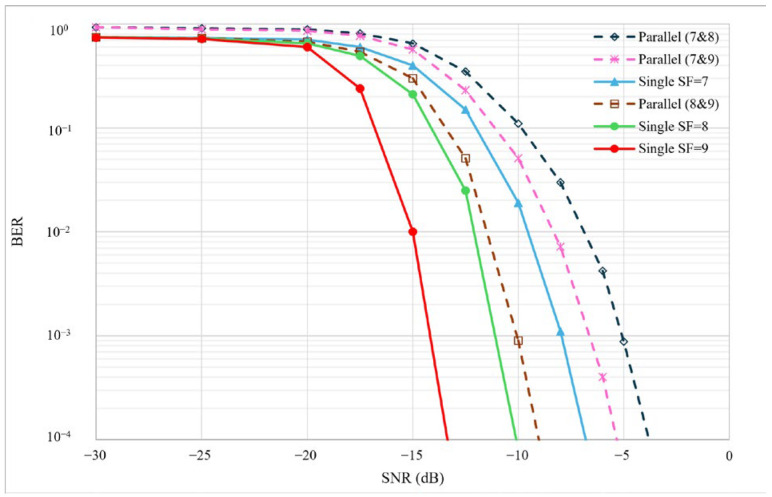
Comparison of BER of our LC (dashed lines) with single transceiver setup (solid lines) using SFs 7, 8, and 9.

Next, we measured the ratio of errored bits to the total image bits to calculate the BER of the LC with a BW of 125 kHz and varying SFs. The experiments are carried out in the same location as earlier in this subsection, with the transmit power altered at the transmitter to have SNR values in the range of [−30, 0] dB. Figure 13 illustrates the BER of different parallel communication setups compared to the analytical BER of a single LoRa physical channel with a BW of 125 kHz and SFs of 7, 8, and 9, as reported in [25]. The solid lines represent the theoretical BER of single LoRa communication links in an LoS scenario with the presence of Additive White Gaussian Noise (AWGN).

It is evident that utilizing a lower SF increases the probability of error in LoRa communications. In our proposed parallel setup, higher SF combinations achieve better BER with SF = 8 and 9 lying just above single SF = 8, outperforming single SF = 7. LCs operating on SF = 7 and 9 and SF = 7 and 8, which improve the *T_tx_*, perform worse than a single transceiver on SF = 7 in terms of probability of error. This is reasonable due to the inter-SF interference of a parallel communication link and the fact that our results are derived from experimental data rather than theoretical models. As shown in Figure 13, for SNR values greater than −5 dB, the BER remains within acceptable limits, demonstrating the viability of our parallel communication setup.

**Figure 14:**
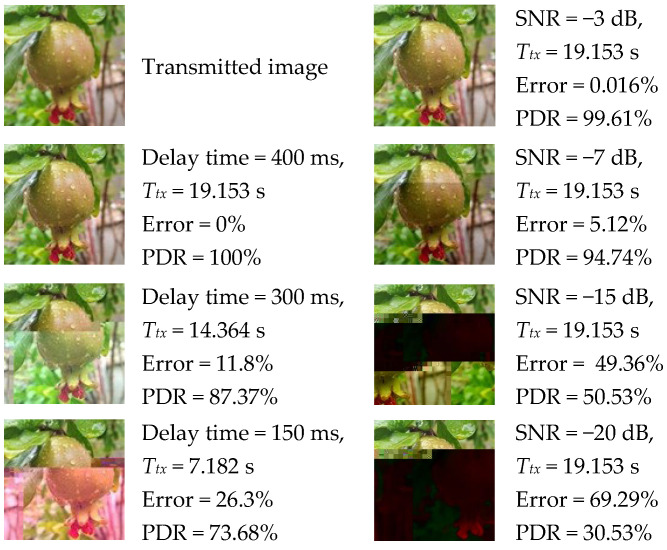
The impact of smaller delay times (left column) and lower SNR values (right column) on the quality of the received image over SF = 7 and 8 and BW = 250 kHz.

**Figure 15:**
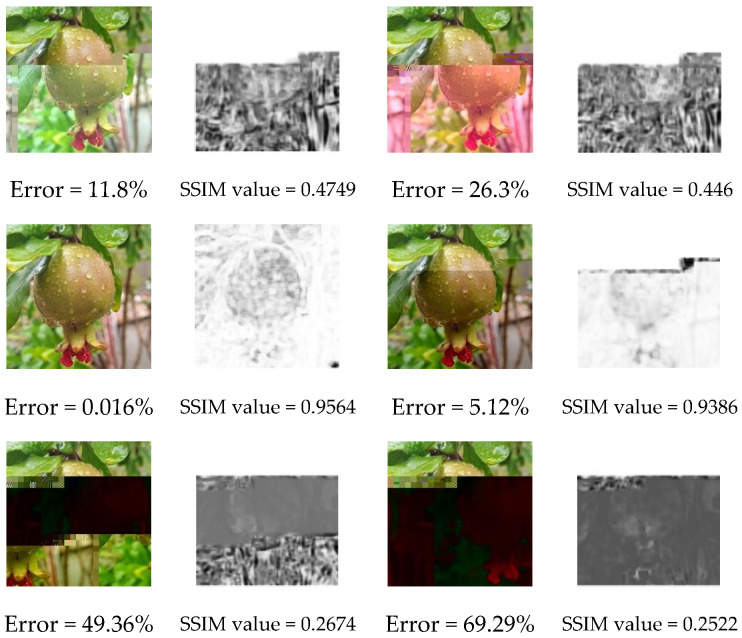
SSIM map and value of the received image with lower delay times and SNR values.

Finally, the quality of the images received through the LC are investigated. Allowing sufficient delay time for packet processing at the receiver along with maintaining an adequate SNR value (higher than −5 dB for LoS communication link), results in high-quality images with BER of approximately 10^−3^ or less. However, reducing delay times to minimize the total *T_tx_* or maintaining a lower signal power to reduce energy consumption will compromise image quality by increasing the probability of errors in the received data. Figure 14 illustrates the impact of utilizing delay times shorter than those derived from our experiments (left column) and the effect of SNR values below −5 dB (right column) on the quality of the received image for the parallel setup operating on SFs 7 and 8 and BW = 250 kHz. The quality of the received image is measured in terms of the ratio of bits received in error to the total number of bits (Error) and packet delivery rate (PDR). Additionally, the Structural Similarity Index Measure (SSIM) of the received image under different delay times and SNR values is calculated. Figure 15 presents the SSIM maps for those received images where lighter gray corresponds to higher similarity (SSIM values closer to 1) and darker gray indicates lower similarity (SSIM values closer to 0). The reference image for this metric is the transmitted image.

Figure 14 and Figure 15 highlight the tradeoff between the quality of the image received and the *T_tx_* or SNR. Depending on the application, if a certain amount of error can be tolerated, the transmission can take less time or less power can be spent on the transmitter amplifier.

In the end, we have compared our proposed algorithm with other LoRa capacity improvement schemes. This comparison is reported in Table 5. ICS-CSS and SSK-ICS improved LoRa capacity by changing the existing CSS modulation. Due to the change in modulation, these schemes may not be compatible with existing LoRa applications until chip-level implementations are available. Additionally, while these modulation changes can increase the BR by up to 42%, our proposed algorithm significantly outperforms them, achieving a 73.5% improvement in BR. TDM-LoRa [10] modulation doubled the data rate, increasing the BER at lower SF (SF = 7), whereas proposed algorithm improves the data rate up to 73.5% using the SF = 7 and 8. TDM-LoRa has modified the CSS modulation and is incompatible with the existing LoRa physical layer. The network access protocol proposed by [13] utilizes multiple SFs across different clusters to equalize transmission time.

Cantor [14] and EWS [15] enhanced the existing LoRaWAN MAC by improving the Packet Reception Rate (PRR) or Packet Delivery Rate (PDR). Cantor introduced a parametric optimization algorithm, which, while increasing network goodput by up to 70%, may generate higher control traffic. EWS, on the other hand, can suffer from a higher collision rate due to inaccurate distance calculations at low RSSI levels. Unlike the CSMA (Carrier Sense Multiple Access) used in LoRaWAN, the proposed algorithm mitigates collisions by employing Listen Before Talk (LBT) and utilizing multiple SFs, instead of RF channels, for parallel transmissions. However, the implementation of Cantor and EWS might not require any hardware modifications, depending on the processing and energy demands. MIMO-LoRa [16] enables the reception of multiple signals with different SFs transmitted in parallel by the same EDs, utilizing SNR and transmit power optimization without needing synchronization. In contrast, the proposed algorithm requires synchronization within the same ED, which adds complexity to the algorithm. While MIMO-LoRa is not suitable for low SFs, the proposed LRA algorithm is optimized for these conditions. However, the proposed algorithm may face challenges with higher SFs, due to increased inter-SF interference. Additionally, as a non-LoRaWAN protocol, the LRA may lack compatibility with existing networks built on LoRaWAN.

## 5. Conclusions

In this study, we addressed the challenge of image transmission using LoRa technology, which is typically constrained by its low data rate. To overcome this limitation, we proposed a resource allocation algorithm for LoRa (LRA) that leverages the quasi-orthogonality of spreading factors (SFs) to enable parallel transmissions of larger data sizes using multiple LoRa modules. To achieve this, we developed a specially designed end device (DED) that incorporates two LoRa modules connected to a single controller unit, minimizing hardware complexity. The LRA algorithm is introduced to assign data packets to the transmitters based on their operating SF in a synchronized manner. Furthermore, we examined the transmission time (*T_tx_*) across different SFs of our logical channel (LC) to optimize the number of packets transmitted per parallel transmission round. We performed a two-phase experimental study to demonstrate the feasibility of the parallel transmissions using different parameters and to examine the performance of our proposed algorithm in terms of *T_tx_*, BER, data rate, energy consumption, and the quality of the received image. The results indicate that our parallel setup significantly improves data rate and *T_tx_* within the existing LoRa technology.

Considering the LRA algorithm in densely deployed LoRa networks to assess scalability would be our next focus to further assess its performance. To achieve this, machine-learning-based approaches for selecting SFs for the DEDs could prove beneficial.

Furthermore, Since LoRa signals with identical SFs can significantly interfere with each other, a coding scheme may exist allowing the receiver to distinguish and successfully demodulate these signals. This could open the door to parallel transmissions on the same SF, greatly boosting throughput. Finding such a coding system would be the subject of future research in this area.

## Figures and Tables

**Table 1:** ToA values of a 240-byte packet for different BWs and SFs.

SF	BW = 500 kHz		BW = 250 kHz		RBRI (%)
	ToA (ms)	BR (kbps)	ToA (ms)	BR (kbps)	
6	52.1	37.5	104.2	18.75	71
7	92.2	21.87	184.4	10.935	75
8	164.0	12.5	327.9	6.25	78
9	292.1	7.03	584.2	3.515	80
10	533.0	3.906	1066.0	1.953	82
11	963.6	2.148	1927.2	1.074	83
12	1804.3	1.172	3608.6	0.586	-

**Table 2:** *T_tx_* results for parallel transmission of a 24 kB image over the LC on SFs 7 and 8.

BW (kHz)	SF	Delay (ms)	RSSI (dBm)	*T_tx_* (s)	*E_tx_* (J)	*E_rx_* (J)	*E_total_* (J)
125	7	700	−41	32.971	9.46	1.25	10.71
	8		−35				
250	7	400	−41	19.153	5.49	0.727	6.217
	8		−37				
500	7	250	−47	12.255	3.52	0.465	3.985
	8		−37				

**Table 3:** *T_tx_* results for parallel transmission of a 24 kB image over the LC on other SF combinations.

BW (kHz)	SF	Delay (ms)	RSSI (dBm)	*T_tx_* (s)	*E_tx_* (J)	*E_rx_* (J)	*E_total_* (J)
125	7	950	−40	48.776	13.34	1.85	15.85
	9		−31				
	7	2000	−41	94.206	27.05	3.57	30.62
	10		−26				
	8	1450	−38	73.519	21.11	2.79	23.9
	9		−32				
	8	2500	−37	122.742	35.24	4.69	39.93
	10		−25				
250	7	600	−44	26.434	7.59	1.00	8.59
	9		−30				
	7	900	−44	42.126	12.09	1.60	13.69
	10		−22				
	8	870	−37	38.213	10.97	1.45	12.42
	9		−32				
	8	1150	−35	53.12	15.25	2.06	17.31
	10		−21				
500	7	350	−42	16.758	4.81	0.64	5.45
	9		−31				
	7	500	−44	23.675	6.80	0.89	7.69
	10		−25				
	8	460	−35	22.45	6.44	0.85	7.29
	9		−31				
	8	650	−37	29.971	8.60	1.14	9.74
	10		−25				

**Table 4:** *T_tx_* results of the same image transmission over a single transceiver setup with different BWs.

BW (kHz)	SF	Delay (ms)	RSSI (dBm)	*T_tx_* (s)	*E_tx_* (J)	*E_rx_* (J)	*E_total_* (J)
125	7	550	−45	57.205	16.42	2.17	18.59
250	7	300	−40	29.138	8.37	1.10	9.47
500	7	200	−41	19.816	5.69	0.75	6.44

**Table 5:** Comparison of our proposed LRA algorithm with the other LoRa capacity improvement schemes.

Scheme	ICS-CSS [6]	SSK-ICS [7]	TDM-LoRa [10]	[13]	Cantor [14]	EWS [15]	MIMO-LoRa [16]	LRA Algorithm
Technique	Modulation			Dynamic SF allocation	Optimization algorithm		Application layer	Physical and MAC layer
LoRa orthogonality	Similar to traditional CSS		Quasi-orthogonal on radio channel	Similar to traditional CSS		SF orthogonality		SF orthogonality
Compatibility	Incompatible to LoRa-PHY			Compatible with existing LoRaWAN-MAC			Incompatible with LoRaWAN	Hardware compatible with LoRa-PHY, incompatible with LoRaWAN MAC
Implementation	Need HW redesign			No HW redesign required, need SW implementation			May need HW redesign	No HW redesign needed. Simple SW implementation
Limitations	Incompatible with the existing LoRa technology due to change in modulation technique			Bottleneck for nodes closer to the GW for multi hop network due to use of higher SF	Calculation and control message overhead for the optimization algorithm	Require location data from GPS or derive from RSSI	Performs better at higher SFs (10, 11, 12)	Not suitable for combination of higher SF with lower (e.g., 7 and 12)
Advantages	May not impact the implementation of available LoRaWAN			Compatible with existing LoRaWAN	Compatible with existing LoRaWAN	Increase network size	Increased network coverage area	Easy implementation, suitable for image transmission
Performance Improvement	42% BR gain with 3.39% increase in BER	28.6% BR improvement for the same SF and BW	Doubled the BR, increased BER at lower SF (SF = 7)	62.8% BR improvement at SF = 7	70% BR improvement	18.2% to 55.25% BR improvement	10% to 50% BR improvement at SNR ≤ 10 dB	73.5% (at SF = 7 and 8), 24.9% (at SF = 7 and 9) BR improvement, 42.4% (at SF = 7 and 8), 19.98% (at SF = 7 and 9) ToA improvement

## Data Availability

Data are contained within the article.
